# Peculiarities of the Acetylcholine Action on the Contractile Function of Cardiomyocytes from the Left and Right Atria in Rats

**DOI:** 10.3390/cells11233809

**Published:** 2022-11-28

**Authors:** Xenia Butova, Tatiana Myachina, Raisa Simonova, Anastasia Kochurova, Yakov Bozhko, Michael Arkhipov, Olga Solovyova, Galina Kopylova, Daniil Shchepkin, Anastasia Khokhlova

**Affiliations:** 1 Institute of Immunology and Physiology, Russian Academy of Sciences, Pervomajskaya Str. 106, 620049 Yekaterinburg, Russia; 2 Department of Therapy, Ural State Medical University, Repina Str. 3, 620028 Yekaterinburg, Russia; 3 Institute of Natural Sciences and Mathematics, Ural Federal University, Mira 19, 620002 Yekaterinburg, Russia

**Keywords:** acetylcholine, left and right atria, single cardiomyocytes, sarcomere shortening, actin–myosin interaction, [Ca^2+^]_i_ transients, phosphorylation of sarcomeric proteins

## Abstract

Acetylcholine (ACh) is the neurotransmitter of the parasympathetic nervous system that modulates cardiac function, and its high concentrations may induce atrial fibrillation. We compared the ACh action on the mechanical function of single cardiomyocytes from the left atria (LA) and the right atria (RA). We exposed single rat LA and RA cardiomyocytes to 1, 10, and 100 µM ACh for 10–15 min and measured the parameters of sarcomere shortening–relengthening and cytosolic calcium ([Ca^2+^]_i_) transients during cell contractions. We also studied the effects of ACh on cardiac myosin function using an in vitro motility assay and analyzed the phosphorylation level of sarcomeric proteins. In LA cardiomyocytes, ACh decreased the time to peak sarcomere shortening, time to 50% relengthening, and time to peak [Ca^2+^]_i_ transients. In RA cardiomyocytes, ACh affected the time of shortening and relengthening only at 10 µM. In the in vitro motility assay, ACh reduced to a greater extent the sliding velocity of F-actin over myosin from LA cardiomyocytes, which was accompanied by a more pronounced decrease in phosphorylation of the myosin regulatory light chain (RLC) in LA cardiomyocytes than in RA cardiomyocytes. Our findings indicate that ACh plays an important role in modulating the contractile function of LA and RA, provoking more pronounced changes in the time course of sarcomere shortening–relengthening and the kinetics of actin–myosin interaction in LA cardiomyocytes.

## 1. Introduction

Acetylcholine (ACh) is the neurotransmitter that is synthesized by the parasympathetic nervous system and modulates heart function interacting with cardiomyocyte cholinergic muscarinergic receptors (primarily M2 subtype) [1,2]. ACh is released from the vagal nerve end, which predominantly innervates the atrial conduction system but not the ventricles. However, cardiomyocytes themselves are able to synthesize, transport, store and excrete ACh in the heart, suggesting their involvement in the neuro-myocardial transmission [3]. These findings suggest that cardiomyocytes possess an ACh synthesis system, which is positively modulated by cholinergic stimuli [4].

ACh regulates signaling pathways necessary to maintain electrical activity and Ca^2+^ cycling in the heart [5,6,7]. With a change in the activity of the vagal nerve, in the atria, the ACh concentration changes significantly (e.g., it increases by 9 times at 20 Hz vagal stimulation and by 20 times at 40 Hz stimulation [8]). Both ACh infusion at high concentration and high-level vagal stimulation promote the inducibility of atrial fibrillation (AF) [9,10,11,12]. The arrhythmogenic effects of ACh are related to the ACh-activated K^+^ current (I_K,ACh_), which hyperpolarizes the membrane and shortens action potential duration (APD) in atrial cardiomyocytes facilitating the onset and maintenance of AF by reducing the wavelength for re-entry [11,13]. It was found that cardiomyocytes from the left atrium (LA) have greater densities of M2 receptors and I_K,ACh_, leading to enhanced vulnerability to AF compared to cardiomyocytes from the right atrium (RA) [14,15]. The authors suggested that the different sensitivity of LA and RA myocytes to ACh results in a greater increase in rotor frequency in LA than in RA, an increased LA-to-RA frequency gradient, and increased incidence of wavelet formation during AF.

ACh directly influences the contractility of the atrial myocardium, decreasing the force of atrial preparations and atrial cardiomyocyte shortening [16,17] or provoking positive and negative inotropic changes dependent on the applied concentration [18]. Whereas the different effects of ACh on electrophysiological characteristics in LA and RA have been widely discussed [14,15,19], the differences in mechanical responses of LA and RA to ACh were not examined. ACh via phosphatidylinositol-3-kinase (PI3K)/Akt pathway signaling can affect the phosphorylation level of sarcomeric proteins and the contractile function of cardiomyocytes [20,21]. In this study, we tested the hypothesis that single LA cardiomyocytes are more sensitive to ACh than RA cardiomyocytes. We measured sarcomere shortening–relengthening and cytosolic calcium ([Ca^2+^]_i_) transients in contracting cardiomyocytes from rat LA and RA at different ACh concentrations (1–100 µM). To explore potential mechanisms of ACh-induced differences in contractile responses between LA and RA cardiomyocytes, we studied the effects of ACh on the characteristics of actin–myosin interaction using an in vitro motility assay and analyzed a phosphorylation level of sarcomeric proteins.

## 2. Materials and Methods

All experiments were carried out according to Directive 2010/63/EU and approved by the Animal Care and Use Committee of the Institute of Immunology and Physiology of RAS. Male Wistar rats (aged 10 weeks, m = 250–300 g) were maintained under standard conditions (12 h light/dark cycle with ad libitum access to water and food). Before the heart isolation, the animals were deeply anesthetized with an intramuscular injection of 0.3 mL/kg Zoletil 100^®^ (Virbac, Carros, France) and 1 mL/kg Xylazine 2% (Alfasan, Woerden, Netherlands), heparinized with 5000 IU/kg sodium heparin (Ellara, Russia) to prevent a blood clotting in coronary vessels, and rapidly euthanized by exsanguination. Unless otherwise noted, all chemicals and reagents were purchased from Merck (St Louis, MO, USA).

### 2.1. Atrial Cardiomyocyte Isolation

Single cardiomyocytes from LA and RA were isolated using a combined technique of Langedorff perfusion and intra-chamber injections as described in detail elsewhere [22]. Briefly, the isolated heart was cannulated via the aorta to the Langendorff apparatus and perfused at a rate of 3.0–3.5 mL/min with a sequence of three solutions equilibrated with 100% O_2_. During the procedure, all solutions were oxygenated with 100% O_2_ and maintained at 35.5 °C. The perfusion was started with a heparinized (10 IU/mL) physiological solution (in mM: 140.0 NaCl, 5.4 KCl, 1.2 MgSO_4_, 10.0 HEPES, 20.0 taurine, 5.0 adenosine, 11.1 D-glucose, 1.0 CaCl_2_, pH 7.35) for 5 min after the heart started regular beating (80–110 beat/min). Then, perfusion was switched to a nominally free Ca^2+^-low Na^+^-high K^+^ solution (in mM: 115.0 NaCl, 14.0 KCl, 1.2 MgSO_4_, 10.0 HEPES, 20.0 taurine, 5.0 adenosine, 11.1 D-glucose, 0.03 EGTA, 0.05 CaCl_2_, pH 7.25) for 10 min after the heart has stopped beating. Next, the heart was perfused for 10–15 min with a EGTA-free-low Na^+^-high K^+^ enzyme solution, containing 0.8 mg/mL collagenase II (~305 IU/mL; Worthington Biochemical, Lakewood, NJ, USA), 0.06 mg/mL protease XIV (~3.5 IU/mL), and 0.025 mM CaCl_2_ (pH 7.25). Simultaneously with the Langedorff perfusion, the atria were injected with an enzyme solution containing 1.0 mg/mL collagenase II and 0.06 mg/mL protease XIV. After the appearance of viscous drops at the heart apex, the heart was removed from the Langendorff apparatus, and atria were transferred to a Petri dish for the intra-atrial injections with an enzyme solution (0.9 mg/mL collagenase II and 0.06 mg/mL protease XIV) for ≈25 min. Afterward, LA and RA were separated by cutting along the interatrial septum, cut into small pieces, re-suspended with an EGTA-free-low Na-high K^+^ enzyme solution supplemented with Bovine Serum Albumin (5 mg/mL), and gradually adjusted to an increasing extracellular Ca^2+^ concentration (0.1–1.0 mM). The final cell pellet was stored in a modified Tyrode solution (140.0 mM NaCl, 5.4 mM KCl, 1.8 mM CaCl_2_, 1.0 mM MgSO_4_, 10.0 mM HEPES, 11.1 mM D-glucose, pH 7.35) at room temperature (22 ± 2 °C) and used within 4–6 h. Isolated cardiomyocytes were kept at rest for at least 30 min before being used in experiments.

### 2.2. Incubation of Atrial Cardiomyocytes with Acetylcholine

The stock solution (1 mM) of acetylcholine chloride (AChCl, ≥99% purification) was prepared daily and diluted in a Tyrode solution to the desired concentration: 1, 10, and 100 µM. For measurements of sarcomere shortening–relengthening and [Ca^2+^]_i_ transients, resting (non-stimulated) cardiomyocytes were incubated at 30 ± 1 °C in an ACh-containing solution for 8 min, and then, cells were field-stimulated at 1 Hz for at least 2 min for equilibration before functional measurements. The duration of incubation with ACh for each cardiomyocyte did not exceed more than 15 min. Cardiomyocytes from the control group were exposed to the same protocol in a Tyrode solution without ACh.

To study actin–myosin interactions and sarcomeric protein phosphorylation, atrial cardiomyocytes from each heart were separated into ACh-treated (10–15 min of incubation) and control groups (“individual” control for each ACh concentration), and then cardiac myosin and protein samples for gel staining were prepared.

### 2.3. Measurements of Sarcomere Shortening–Relengthening in Single LA and RA Cardiomyocytes

Sarcomere shortening–relengthening (steady-state conditions) during mechanically non-loaded contractions of LA and RA cardiomyocytes at each ACh concentration was measured using the IonOptix system (IonOptix Corporation, Milton, MA, USA). Only spindle-shaped cardiomyocytes with well-defined sarcomere striations were examined. The average sarcomere length (SL) was calculated from the intensity profile derived on the sarcomere striation pattern in a selected narrow region on the cell surface using Ion Wizard software (IonOptix Corporation, Milton, MA, USA). All experiments on single cardiomyocytes were performed at 1 Hz and 30 ± 1 °C.

The following parameters were further used for the statistical analysis: end-diastolic SL (EDSL), SL shortening amplitude (SLs = EDSL minus end-systolic SL), fractional SL shortening amplitude (FSL_S_ = SL_S_/EDSL × 100%), time from the start of SL shortening to peak shortening (time to peak SL shortening, TTP_S_), time from peak shortening to 50% sarcomere relengthening (TTR_50_), and maximum velocities of sarcomere shortening and relengthening (v_S_ and v_R_).

### 2.4. Measurements of [Ca^2+^]_i_ Transients in Single LA and RA Cardiomyocytes

[Ca^2+^]_i_ transients in separate groups of mechanically non-loaded LA and RA cardiomyocytes were recorded using a LSM 710 scanning confocal system and Zen 2010 software (Carl Zeiss, Jena, Germany). For imaging of [Ca^2+^]_i_ transients, cardiomyocytes were incubated with 1.7 µM Fluo-8AM (AAT Bioquest, Sunnyvale, CA, USA) and 0.1% Pluronic^®^ F-127 (AAT Bioquest, Sunnyvale, CA, USA) in darkness for 20 min at room temperature and then washed with a modified Tyrode solution. The Fluo-8AM was excited optically using Ar-laser at 488 nm. The intensity of emitted fluorescence was collected at 493–575 nm from a selected narrow region on the cell surface (3 pixels high, 200 pixels length). The change in the fluorescence signal (ΔF/F_0_, where F_0_ is the minimal fluorescence intensity average measured between 1 Hz contractions at the diastolic phase of [Ca^2+^]_i_ transients) was calculated and used as an index of [Ca^2+^]_i_ change.

The amplitude of [Ca^2+^]_i_ transients (CaT), time from the start of [Ca^2+^]_i_ increase to peak systolic [Ca^2+^]_i_ (time to peak [Ca^2+^]_i_ transient, TTP_Ca_), and the time from TTP_Ca_ to 50% decay of [Ca^2+^]_i_ transient (TTD_50_) were calculated using custom-made software EqapAll 6 [23] and then were used for the statistical analysis.

### 2.5. Extraction of Sarcomeric Proteins and Phosphorylation Analysis

For in vitro motility assay experiments, cardiac myosin was extracted from LA and RA cardiomyocytes according to [24] with modifications. F-actin was obtained from the bovine left ventricle [25].

Protein phosphorylation was analyzed using a 12% SDS-PAGE with Pro-Q Diamond phosphoprotein staining (Invitrogen, Eugene, OR, USA). SYPRO Ruby (Invitrogen, Eugene, OR, USA) staining was used to estimate the total amount of protein. Protein samples and gel staining were prepared according to the manufacturer’s manual. The gel was scanned on the ChemiDoc MP Imaging System (Bio-Rad, Hercules, CA, USA), and band densities were determined with Image Lab 5.2.1 software (Bio-Rad, Hercules, CA, USA). A level of protein phosphorylation was expressed as a ratio of the Pro-Q Diamond intensity to the SYPRO Ruby intensity.

### 2.6. In Vitro Motility Assay

The in vitro motility assay experiments were performed as described in detail previously [26]. Briefly, 500 µg/mL myosin in an AB buffer (in mM: 25 KCl, 25 imidazole, 4 MgCl_2_, 1 EGTA, and 20 DTT, pH 7.5), containing 0.5 M KCl was loaded into the flow chamber with nitrocellulose inner surface. After 2 min, 0.5 mg/mL BSA was added for 1 min. Furthermore, 50 µg/mL of non-labeled F-actin in the AB buffer with 2 mM ATP was added for 5 min. TRITC-phalloidin labeled F-actin at a concentration of 10 nM (by G-actin) was added for 5 min. Unbound filaments were washed out with AB buffer. Finally, the chamber was washed with AB buffer containing 0.5 mg/mL, BSA, oxygen scavenger system, 20 mM DTT, 2 mM ATP, 0.5% methylcellulose. The experiments were performed at 30 °C. The sliding velocities of 100 actin filaments per experiment were measured using the GMimPro software [27].

### 2.7. Statistical Analysis

All experimental data were collected with Excel 16 (Microsoft Corp, Redmond, WA, USA) and analyzed using GraphPrism 8.0 software (Origin Lab, Northampton, MA, USA). Data are expressed as median and interquartile range. For parametric statistical analyses, data were distributed normally (checked using a Shapiro–Wilk normality test). The parameters between the LA and RA control groups (0 µM ACh) were compared using the t-test or Mann–Whitney U-test. If the variance was similar between the groups (checked using Bartlett’s test), statistical comparisons of multiple groups (0, 1, 10, and 100 µM ACh) were performed using one-way ANOVA followed by Holm–Sidak’s multiple comparisons test. If the variance between analyzed groups was different, Brown–Forsythe and Welch ANOVA tests followed by Dunnett’s multiple comparisons test were performed. For non-parametric statistical analyses of multiple groups, we performed a Kruskal–Wallis test with Dunn’s multiple comparisons test. The velocity of F-actin and sarcomeric protein phosphorylation for each ACh concentration was compared to the individual control values by Mann–Whitney U-test. A *p*-value of <0.05 was considered to indicate a significant difference between groups.

## 3. Results

### 3.1. The Action of Acetylcholine on Sarcomere Shortening–Relengthening in Single LA and RA Cardiomyocytes

**Figure 1:**
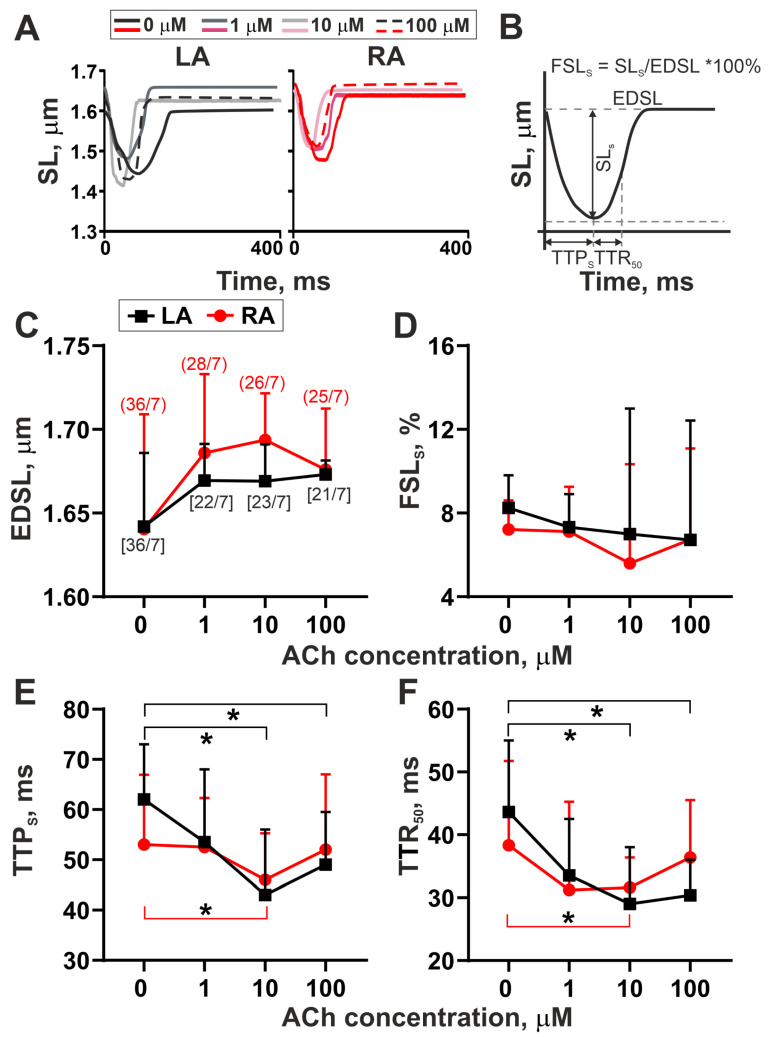
Dose–response curves of the amplitude and time course parameters of sarcomere shortening–relengthening in single mechanically non-loaded LA and RA cardiomyocytes to 1–100 µM ACh. (**A**) Representative recordings of time-dependent sarcomere length (SL) changes in LA and RA cardiomyocytes from the control group (0 µM) and after incubation with ACh for 10–15 min. (**B**). Analyzed parameters derived from SL change signal. (**C**) End-diastolic SL (EDSL). (**D**) Fractional SL shortening amplitude (FSL_S_ = SL_S_/EDSL × 100%). (**E**) Time to peak SL shortening (TTP_S_). (**F**) Time from peak shortening to 50% sarcomere relengthening (TTR_50_). The number of n cells from N hearts (n/N) is shown in square brackets (LA) or parentheses (RA) in the (**C**) panel. Values are plotted as medians with interquartile ranges. * *p* < 0.05: 1, 10, and 100 µM ACh compared to the control group (0 µM ACh); one-way ANOVA (EDSL), Brown–Forsythe and Welch ANOVA tests (FSL_S_), and Kruskal–Wallis test (TTP_S_ and TTR_50_).

Representative traces of mechanically non-loaded sarcomere shortening–relengthening in single LA and RA cardiomyocytes in the control groups and after 10–15 min incubation of cells with ACh are shown in Figure 1A. The examined parameters are depicted in Figure 1B. We did not find differences in the parameters between LA and RA cardiomyocytes in the control groups (*p* > 0.1, t-test or Mann–Whitney U-test). ACh did not affect EDSL in both LA and RA cardiomyocytes (Figure 1C). Neither absolute SL shortening amplitude (SL_S_) nor fractional SL shortening amplitude (FSL_S_) changed under ACh application for each cell type (*p* = 0.9, one-way ANOVA or Brown–Forsythe and Welch ANOVA tests, Figure 1D and Figure S1C in Supplementary Materials).

ACh at 10 µM decreased time to peak shortening (TTP_S_) and time to 50% sarcomere relengthening (TTR_50_) in both cell types (Figure 1E,F), while it did not affect significantly maximum velocities of sarcomere shortening and relengthening (Figure S1C,D in Supplementary Materials). TTP_S_ and TTR_50_ were reduced by ~40% and 42%, respectively, in LA cardiomyocytes and by ~ 62 and 32%, respectively, in RA cardiomyocytes (10 µM ACh, *p* < 0.05, Kruskal–Wallis test, Figure 1E,F). In contrast to RA cardiomyocytes, in LA cardiomyocytes, TTP_S_ and TTR_50_ were also decreased at 100 µM ACh, indicating that LA cardiomyocytes are more sensitive to ACh application in a wider ACh concentration range.

Thus, the short-time incubation of atrial cardiomyocytes with ACh affected the time course parameters in LA cardiomyocytes in a wide range of ACh concentrations without the influence on the amplitude of sarcomere shortening.

### 3.2. The Action of Acetylcholine on [Ca^2+^]_i_ Transients in Single LA and RA Cardiomyocytes

**Figure 2:**
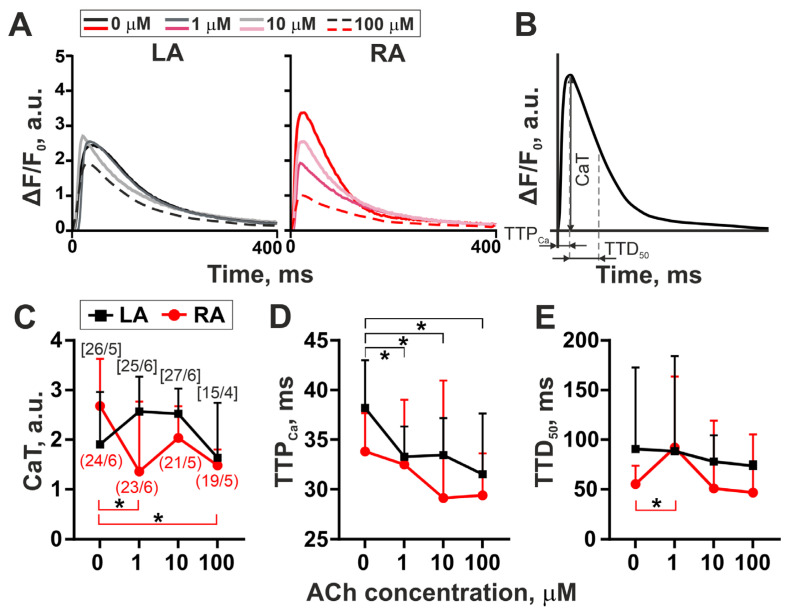
Dose–response curves of the amplitude and time course parameters of [Ca^2+^]_i_ transients in single mechanically non-loaded LA and RA cardiomyocytes to ACh (1–100 µM). (**A**) Representative recordings of [Ca^2+^]_i_ transients in LA and RA cardiomyocytes from the control group (0 µM) and after incubation with 1, 10, and 100 µM ACh for 10–15 min. (**B**) Analyzed parameters derived from F/F_0_ ([Ca^2+^]_i_ changes) signal. (**C**) Amplitude of [Ca^2+^]_i_ transient (CaT). (**D**) Time to peak [Ca^2+^]_i_ transient (TTP_Ca_). (**E**) Time to 50% decay of [Ca^2+^]_i_ transients (TTD_50_). The number of n cells from N hearts (n/N) is shown in square brackets [LA] or parentheses (RA) in the (**C**) panel. Values are plotted as medians with interquartile ranges. * *p* < 0.05: 1, 10, and 100 µM ACh compared to the control group (0 µM ACh); Kruskal–Wallis test for all parameters.

Representative traces depicting the effects of 1, 10, and 100 µM ACh on [Ca^2+^]_i_ transients in single LA and RA cardiomyocytes and the analyzed parameters are shown in Figure 2A,B. In the control groups, RA cardiomyocytes had greater amplitudes of [Ca^2+^]_i_ transients (CaT) and shorter time to peak [Ca^2+^]_i_ transients (TTP_Ca_) and time to 50% decay of [Ca^2+^]_i_ transients (TTD_50_) (*p* = 0.0343, *p* = 0.0212 and *p* = 0.0481, respectively, Mann–Whitney U-test). In RA cardiomyocytes, 1 and 100 µM ACh decreased CaT by ~43% compared to control values (*p* = 0.0009 and *p* < 0.0001, respectively; Kruskal–Wallis test, Figure 2C), while 10 µM ACh did not affect CaT (*p* = 0.2436). In LA cardiomyocytes, ACh did not change CaT.

ACh had an ambiguous influence on the time course parameters of [Ca^2+^]_i_ transients in atrial cardiomyocytes. In LA cardiomyocytes, ACh at all concentrations decreased TTP_Ca_ by ~30–38%, compared to the control group (*p* < 0.05, Kruskal–Wallis test, Figure 2D) but did not affect TTD_50_ (*p* > 0.99). In RA cardiomyocytes, ACh did not influence TTP_Ca_ (*p* > 0.99), while 1 µM ACh prolonged TTD_50_ by ~50% compared to the control group values (*p* < 0.05, Kruskal–Wallis test, Figure 2E).

Thus, ACh-induced changes in [Ca^2+^]_i_ transients differed for LA and RA cardiomyocytes. ACh decreased CaT and increased TTD_50_ in RA cardiomyocytes and decreased TTP_Ca_ in LA cardiomyocytes.

### 3.3. The Action of Acetylcholine on the Actin–Myosin Interaction in LA and RA Cardiomyocytes

**Figure 3:**
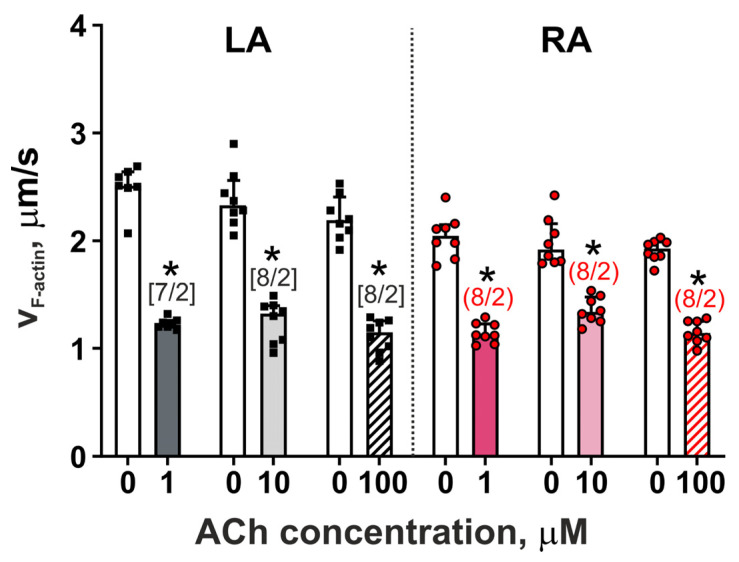
The acute effects of ACh on the sliding velocity of F-actin (v_F-actin_) over myosin from LA and RA cardiomyocytes in an in vitro motility assay. The number of n samples from N hearts (n/N) is shown in square brackets [LA] or parentheses (RA). Data are presented as dots (values) and medians (boxes) with interquartile range (bars). The same hearts were used for ACh groups and individual controls. * *p* < 0.05: 1, 10, and 100 µM ACh compared to the individual control group (0 µM ACh), Mann–Whitney U-test.

The acute effects of ACh on actin–myosin interaction were explored by analyzing the sliding velocity of F-actin over myosin from LA and RA cardiomyocyte suspensions in an in vitro motility assay (v_F-actin_). In the control group, v_F-actin_ over myosin from LA cardiomyocytes was higher compared to v_F-actin_ over myosin from RA cardiomyocytes (*p* = 0.0030, Mann–Whitney U-test, Figure 3). ACh decreased v_F-actin_ over myosin from both LA and RA cardiomyocytes at each analyzed concentration (*p* < 0.01, Mann–Whitney U-test, Figure 3). For LA cardiomyocytes, ACh decreased v_F-actin_ by ~51%, 43%, and 47%, while for RA cardiomyocytes, ACh reduced v_F-actin_ by ~45%, 30%, and 40% at 1, 10, and 100 µM ACh, respectively.

Thus, LA myosin was more sensitive to ACh application compared to RA myosin, which is consistent with findings obtained in single cardiomyocytes. However, the non-changed velocity of shortening and decreased TTP_S_ obtained in single cardiomyocytes was not associated with the ACh-induced decrease in v_F-actin_.

### 3.4. The Action of Acetylcholine on Sarcomeric Protein Phosphorylation in LA and RA Cardiomyocytes

**Figure 4:**
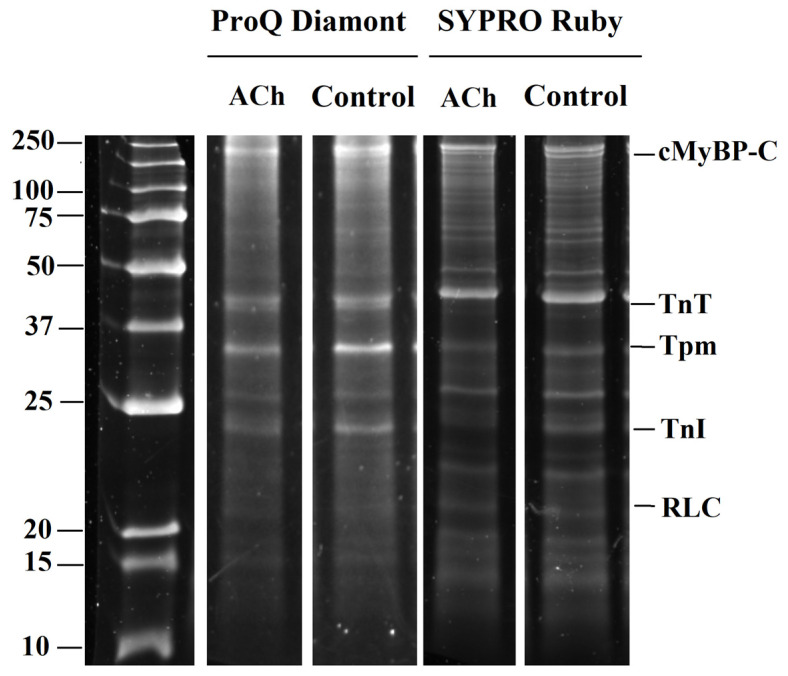
The example of gel electrophoresis of the sarcomeric protein extraction from RA cardiomyocytes of the control group (0 µM) and after incubation with 100 µM acetylcholine (ACh) for 10–15 min. cMyBP-C–cardiac myosin binding protein-C; TnT–troponin T; Tpm–tropomyosin; TnI–troponin I; RLC–myosin regulatory light chain. Phosphorylation was assessed using Pro-Q Diamond and SYPRO Ruby (Invitrogen, Eugene, OR, USA). Precision Plus Protein™ Unstained Standards (Bio-Rad, Hercules, CA, USA) was used as molecular weight markers for protein.

Additionally, to explore potential mechanisms of ACh-induced differences in contractile responses between LA and RA cardiomyocytes, we analyzed changes in the phosphorylation levels of the regulatory light chain of myosin (RLC), cardiac myosin binding protein C (cMyBP-C), troponin T (TnT), troponin I (TnI), and tropomyosin (Tpm) in LA and RA cardiomyocytes (Figure 4).

**Figure 5:**
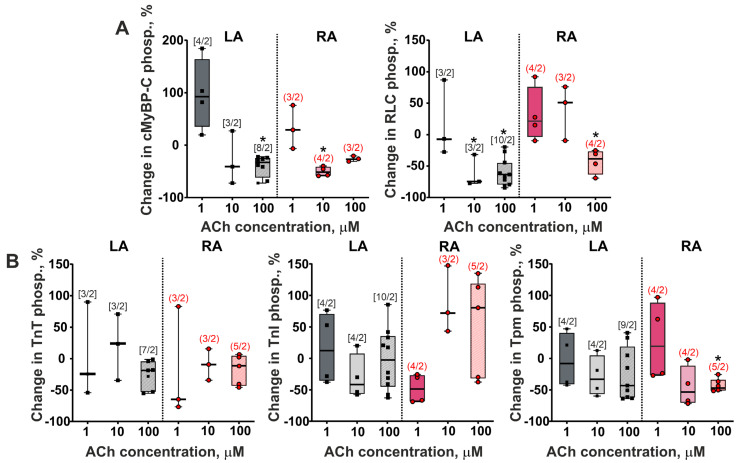
The acute effects of ACh on sarcomeric protein phosphorylation in LA and RA cardiomyocytes. cMyBP-C–cardiac myosin binding protein-C; RLC–myosin regulatory light chain; TnT–troponin T; TnI–troponin I; Tpm–tropomyosin. (**A**) Phosphorylation levels of cMyBP-C and RLC. (**B**) Phosphorylation levels of TnT, TnI, and Tpm. The same hearts were used for ACh groups and individual controls. Phosphorylation is expressed as the ratio of the intensities of protein bands stained with Pro-Q Diamond and SYPRO Ruby, and then calculated as the % change related to the individual control values. The number of n samples from N hearts is shown in square brackets [LA] or parentheses (RA). Data are presented in box and whisker plots, where the boxes are drawn from Q1 to Q3, horizontal lines represent median values and whiskers give the 100% range of the values. * *p* < 0.05: 1, 10, and 100 µM ACh compared to the individual control group (0 µM ACh), Mann–Whitney U-test.

We found that acute ACh application decreased the phosphorylation level of cMyBP-C and RLC in both cell types (Figure 5A). The phosphorylation level of cMyBP-C was decreased by ~52% at 10 µM in RA cardiomyocytes (*p* = 0.0471), and by ~33% at 100 µM in LA cardiomyocytes (*p* = 0.0007, Mann–Whitney U-test). In LA cardiomyocytes, the phosphorylation level of RLC was decreased by ~75% at 10 µM ACh and by ~63% at 100 µM ACh. In RA cardiomyocytes, it was decreased by ~38% at 100 µM ACh (*p* = 0.0435, Figure 5A). TnT and TnI phosphorylation levels did not differ after cell incubation with ACh, while the phosphorylation level of Tpm was decreased only in RA cardiomyocytes by ~47% at 100 µM ACh (*p* < 0.01, Mann–Whitney U-test, Figure 5B).

Thus, short-term incubation of atrial cardiomyocytes with ACh decreased the phosphorylation levels of cMyBP-C and RLC in both LA and RA cardiomyocytes, and decreased the phosphorylation level of Tpm in RA cardiomyocytes.

## 4. Discussion

This study examined the acute effects of ACh on sarcomere dynamics and [Ca^2+^]_i_ transients in rat atrial cardiomyocytes. For the first time, we revealed the distinct mechanical responses of LA and RA cardiomyocytes to ACh application in a range of 1–100 µM. We also showed that ACh differently affects actin–myosin interaction and sarcomeric phosphorylation in LA and RA cardiomyocytes. Our main findings are that: (1) LA cardiomyocytes are more sensitive to the acute application of ACh compared to RA cardiomyocytes, demonstrating greater changes in the time course of sarcomere shortening–relengthening, in time to peak [Ca^2+^]_i_ transients, and in velocity of thin filament sliding on cardiac myosin in the in vitro motility assay; (2) ACh at 1–100 µM does not affect the amplitude of sarcomere shortening, decreases the phosphorylation level of cMyBP-C and RLC in LA and RA cardiomyocytes, and it depresses [Ca^2+^]_i_ transient amplitudes in RA cardiomyocytes.

### 4.1. The Effects of ACh on Sarcomere Dynamics in Atrial Cardiomyocytes

The short-term influence of ACh on cardiomyocytes is inherent for the heart in vivo. The actions of ACh at the muscarinic receptors are quickly terminated by the activity of cholinesterase, which hydrolyzes ACh [28]. However, the first minutes of muscarinic receptor activation are sufficient for the induction of immediate early gene expression by activation of protein kinase C (PKC) [29].

Our study showed that 10–15 min incubation of atrial cardiomyocytes with ACh (1–100 µM) decreased the phosphorylation level of cardiac myosin binding protein-C (cMyBP-C) and myosin regulatory light chain (RLC) in LA and RA cardiomyocytes, depressed [Ca^2+^]_i_ transient amplitudes in RA cardiomyocytes but did not affect the amplitude of sarcomere shortening in both cell types. These results were obtained at a stimulation frequency of 1Hz. To test whether a stimulation frequency may affect mechanical responses of atrial cardiomyocytes to ACh, we recorded sarcomere shortening–relengthening in LA and RA cardiomyocytes contracting at 0.5–1–2–3–4 Hz. We found no effects of ACh (1–100 µM) either on the absolute or fractional sarcomere shortening in both LA and RA cell types (n = 5–11 cells in each group, N = 2 hearts). Note that rodent hearts normally contract at a higher beating frequency (6–8 Hz), and future studies in this area are relevant for understanding the ACh effects.

In contrast, previous studies demonstrated negative inotropic effects of ACh on atrial cardiomyocyte shortening and force amplitude of isolated atrial preparations from guinea pig (0.01–100 μM) and canine hearts (1 µM) [16,17]. In human isolated atria, ACh elicited complex inotropic effects, decreasing the force amplitude at low concentrations (0.001–1 µM) followed by an increase in the force amplitude at high concentrations (1–1000 µM) [18].

One of the important mechanisms regulating atrial contractility is the myocardial production of nitric oxide (NO). ACh stimulates M2 receptors that are coupled to pertussis toxin-sensitive Gi proteins, and this stimulation via the PI3K/Akt pathway signaling activates NO production [20,21]. NO inhibits the L-type of Ca^2+^ channels but stimulates sarcoplasmic reticulum (SR) Ca^2+^ release, leading to variable effects on myocardial contractility [30]. A study on rat isolated atria showed that the activation of atrial M2 receptors by carbachol promoted the gene expression of neuronal nitric oxide synthase (nNOS) and endothelial nitric oxide synthase (eNOS) [31]. The authors suggested that nNOS may mediate negative inotropic effects in atria, whereas eNOS acts as a positive cardiac regulator. Possibly, distinct contributions of nNOS and eNOS to Ach-induced changes in contractility might be dependent on ACh concentrations and mammalian species that may contribute to data inconsistency. Further studies in this area are required.

ACh has been indicated to facilitate ROS generation in atrial cardiomyocytes by sequentially activating Akt and NOS [32]. ROS activates redox-regulated signaling enzymes (e.g., protein kinase C, and protein kinase A, PKC and PKA), resulting in changes in protein phosphorylation [33]. We found that ACh reduced cMyBP-C and RLC phosphorylation in LA and RA cardiomyocytes (Figure 5), which may result in the impaired contractility of cardiomyocytes [34,35].

### 4.2. Differing Sensitivity of LA and RA Cardiomyocytes to Acute ACh Application

We found that ACh had a greater effect on the time course parameters of sarcomere shortening–relengthening and the kinetics of actin–myosin interaction in LA cardiomyocytes than in RA cardiomyocytes. In LA cardiomyocytes, the time to peak of sarcomere shortening (TTP_S_) and time to 50% of sarcomere relengthening (TTR_50_) were decreased at 10 and 100 µM ACh compared to the control values, while in RA cardiomyocytes, these characteristics were decreased only at 10 µM (Figure 1). A decrease in TTP_S_ in LA cardiomyocytes after ACh application was accompanied by reduced time to peak [Ca^2+^]_i_ transient (TTP_Ca_, Figure 2). Previous studies have shown that densities of M2 receptor, Kir3.1 and Kir3.4 subunits and I_K,ACh_ current are significantly higher in LA cardiomyocytes than in RA cardiomyocytes [14,15]. The stimulation of M2 receptors by ACh results in the dissociation of Gi proteins in Gαi and Gβγ subunits, with the latter activating I_K,ACh_ channels that induces APD shortening [1,2]. In LA cardiomyocytes, where probably more M2 receptors are activated, ACh induces more pronounced APD shortening compared to RA cardiomyocytes due to a greater repolarizing I_K,ACh_ current activated by Kir3.1 and Kir3.4 subunits [14]. Thus, an ACh-induced shortening in APD in LA cardiomyocytes may cause a decrease in [Ca^2+^]_i_ transient duration leading to pronounced shortening in TTP_S_ in a wide range of ACh concentrations.

We found that LA and RA cardiomyocytes in the control group had different parameters of [Ca^2+^]_i_ transients. Faster [Ca^2+^]_i_ transients observed in RA cardiomyocytes compared to LA cardiomyocytes to higher SERCA2a expression in RA than in LA [36]. In LA cardiomyocytes, we did not find the significant effects of ACh on time to 50% decay of [Ca^2+^]_i_ transients (TTD_50_) and [Ca^2+^]_i_ transient amplitudes (CaT). In contrast, in RA cardiomyocytes, ACh non-monotonically reduced CaT (1, 100 µM) and prolonged TTD_50_ (1 µM) (Figure 2). The changes in the parameters of [Ca^2+^]_i_ transients in RA cardiomyocytes may be caused by ACh-induced inhibition of intracellular cyclic adenosine monophosphate (cAMP) signaling [37,38]. After the ACh-induced dissociation of the coupled G protein, Gαi subunits of M2 receptor inhibit adenylate cyclase, resulting in a decrease in the cAMP level. The decrease in cAMP may depress L-type Ca^2+^ current (I_CaL_) and SR Ca^2+^ release [17,37,38]. It has been shown that ACh significantly decreases CaT and prolongs TTD_50_ in rabbit sinoatrial nodal cells by reducing the cAMP-PKA-dependent phosphorylation of ryanodine receptors and phospholamban, leading to the reduced open probability of ryanodine receptors and slower SERCA2a-mediated intracellular Ca^2+^ re-uptake into the SR [37].

ACh decreased the sliding velocity of F-actin over myosin from both types of cardiomyocytes (v_F-actin_) with a greater % decrease in LA cells (Figure 3). A previous study using the in vitro motility assay showed that porcine myosin containing phosphorylated RLC increased actin sliding velocity [39]. In our study, the level of RLC phosphorylation was decreased to a greater extent in LA cardiomyocytes than in RA cardiomyocytes compared to the control group values. Thus, a greater effect of ACh on the RLC phosphorylation level in a wider range of ACh concentrations may contribute to more pronounced changes in v_F-actin_ for LA than for RA cardiomyocytes. The inconsistency between the effects of ACh on the kinetics of actin–myosin interaction obtained in the in vitro motility assay and on the kinetics of sarcomere shortening in intact single cardiomyocytes can be explained by a few reasons. We studied the effects of ACh on cardiac myosin only using F-actin because the number of cardiomyocytes from cell suspension was not sufficient for the extraction of native thin filaments to be used in the in vitro motility assay. In addition, in the in vitro motility assay experiments, the kinetics of calcium is not taken into account.

## 5. Conclusions

We found that LA cardiomyocytes are more sensitive to the acute application of ACh compared to RA cardiomyocytes showing more prominent changes in the time course of sarcomere shortening–relengthening and in the functional properties of cardiac myosin. The obtained differences may be associated with the LA–RA gradient of M2 receptor density and ACh-induced differences in signaling pathways that affect [Ca^2+^]_i_ transients and sarcomeric protein phosphorylation in LA and RA cardiomyocytes.

Our results indicate that ACh plays an important role in modulating the contractile function of atrial cardiomyocytes that may contribute to depressed ventricular function under vagal stimulation.

## 6. Limitations

There were several methodological limitations in our study. The result of vagal stimulation could be complex and may cause some inconsistencies between vagal stimulation and acute ACh application effects. We studied direct ACh effects after cell incubation at 1–100 µM for 10–15 min, while different times of incubation and concentrations might yield distinct results. Experiments on isolated LA and RA cardiomyocytes were performed under mechanically unloaded conditions, although the mechanical load may affect LA–RA differences and observed findings. This issue should be considered while interpreting the results. Experiments were performed using rats, which are known to have significant differences in scale and electrophysiology compared to large mammalian and human hearts. Although the main mechanism of sarcomere shortening–relengthening is the same for all mammalian hearts [40], and that rat hearts can produce triggered activity typical for vagally mediated AF [41], we acknowledge this limitation. In addition, an analysis of Ca^2+^-regulating proteins, such as ryanodine receptor, SR Ca^2+^-ATPase, and calmodulin, is also helpful to conciliate data inconsistencies.

## Data Availability

Not applicable.

## Supplementary Materials

The following supporting information can be downloaded at: https://www.mdpi.com/article/10.3390/cells11233809/s1. Figure S1: Dose-response curves of the amplitude and velocities of sarcomere shortening-relengthening in single mechanically non-loaded LA and RA cardiomyocytes to 1–100 μM acetylcholine (ACh); Figure S2. The acute effects of ACh on sarcomeric protein phosphorylation in LA and RA cardiomyocytes. cMyBP-C—cardiac myosin binding protein-C; RLC—myosin regulatory light chain; TnT—troponin T; TnI—troponin I; Tpm—tropomyosin.
