# Supplementary material for: Peculiarities of the Acetylcholine Action on the Contractile Function of Cardiomyocytes from the Left and Right Atria in Rats
Source: Cells. 2022 Nov 28;11(23):3809. doi: 10.3390/cells11233809 (PMC9737865; doi:10.3390/cells11233809)
Supplement: Supplementary file 1 [file cells-11-03809-s001.zip › cells-2013117-supplementary.pdf]

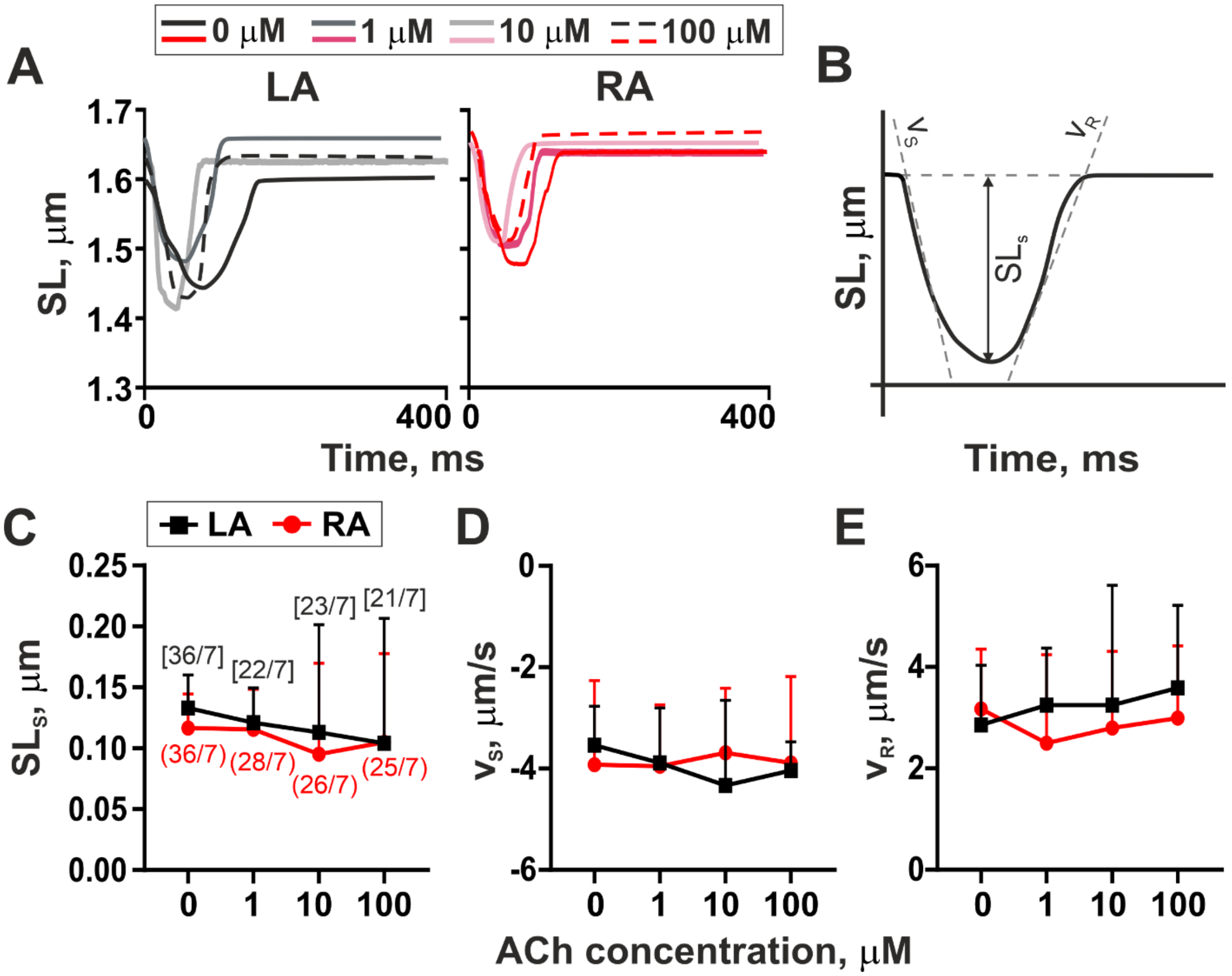

Figure S1. Dose-response curves of the amplitude and velocities of sarcomere shortening-relengthening in single mechanically non-loaded LA and RA cardiomyocytes to 1–100 µM acetylcholine (ACh). (A) Representative recordings of time-dependent sarcomere length (SL) changes in LA and RA cardiomyocytes from the control group (0 µM) and after incubation with ACh for 10–15 min. (B). Analyzed parameters derived from SL change signal. (C) Absolute SL shortening amplitude (SL_s). (D) Maximum velocity of sarcomere shortening (v_S). (E) Maximum velocity of sarcomere relengthening (v_R). The number of n cells from N hearts (n/N) is shown in square brackets [LA] or parentheses (RA) in the (C) panel. Values are plotted as medians with interquartile ranges. *p < 0.05: 1, 10, and 100 µM ACh compared to the control group (0 µM ACh); one-way ANOVA (SL_S) and Kruskal-Wallis test (v_S and v_R).

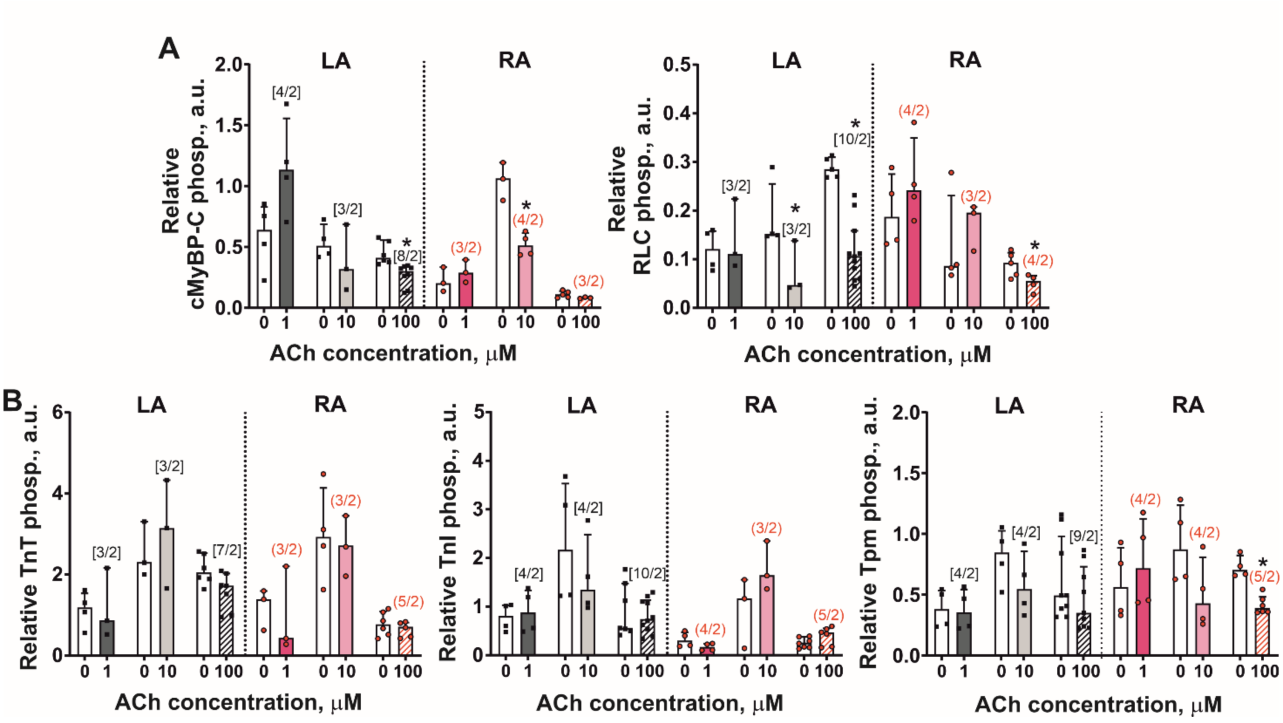

Figure S2. The acute effects of ACh on sarcomeric protein phosphorylation in LA and RA cardiomyocytes. cMyBP-C – cardiac myosin binding protein-C; RLC – myosin regulatory light chain; TnT – troponin T; TnI – troponin I; Tpm – tropomyosin. (A) Phosphorylation levels of cMyBP-C and RLC. (B) Phosphorylation levels of TnT, TnI, and Tpm. Phosphorylation is expressed as the ratio of the intensities of protein bands stained with Pro-Q Diamond and SYPRO Ruby. The same hearts were used for ACh groups and individual controls (stained together with ACh groups). The number of n samples from N hearts is shown in square brackets [LA] or parentheses (RA). Data are presented as dots (values) and medians (boxes) with interquartile range (bars). *$p < 0.05$: 1, 10, and 100 μM ACh compared to the individual control group (0 μM ACh), Mann-Whitney U-test.
